# Addition of a second double‐row reduces fragment micromotion and increases fixation strength in suture fixation of a bony Bankart injury: A biomechanical study

**DOI:** 10.1002/jeo2.70881

**Published:** 2026-08-18

**Authors:** Maximilian Hinz, Ayham Jaber, Justin F. M. Hollenbeck, Rio R. Castro, Wyatt H. Buchalter, Matthew T. Provencher, Peter J. Millett

**Affiliations:** ^1^ Department of BioMedical Engineering Steadman Philippon Research Institute Vail Colorado USA; ^2^ Center for Musculoskeletal Surgery, Charité—Universitätsmedizin Berlin Corporate Member of Freie Universität Berlin and Humboldt‐Universität zu Berlin Berlin Germany; ^3^ Heidelberg University Hospital Heidelberg Germany; ^4^ The Steadman Clinic Vail Colorado USA

**Keywords:** bony Bankart lesion, glenoid rim fracture, shoulder arthroscopy, shoulder dislocation, shoulder instability

## Abstract

**Purpose:**

The purpose of the present study was to evaluate micromotion and failure load when the bony Bankart bridge (BBB) is performed with one versus two double‐rows. It was hypothesized that the addition of a second double‐row would reduce fragment micromotion and increase ultimate failure load.

**Methods:**

Seven pairs of fresh‐frozen cadaveric shoulders (age: 48 years [22–65 years], sex: 57.1% male) with bony Bankart lesions, with a defect width of 25% of the glenoid diameter, were tested in the present study. In a paired design, one shoulder underwent the BBB with one double‐row, and the contralateral shoulder underwent the BBB with two double‐rows. Micromotion of the bony Bankart fragment was tested by cyclic non‐physiological point loading (5–20 N at 1 Hz for 50 cycles) using a dynamic tensile machine. Optical markers and a three‐dimensional digitizer established fragment and glenoid coordinate systems, enabling precise tracking of translational and rotational displacements of the fragment. This was followed by failure testing, where the fragment was loaded to failure at 0.5 mm/s. Failure load and stiffness were recorded.

**Results:**

Under inferior point loading, the repair with one double‐row demonstrated significantly greater rotation about the *x*‐axis (5.20° [3.01°–5.29°] vs. 1.64° [0.94°–2.40°], *p* = 0.016, *q* = 0.047) and translation along the *z*‐axis (2.78 mm [1.81–4.20 mm] vs. 1.06 mm [0.51–1.51 mm], *p* = 0.016, *q* = 0.047) compared with the repair with two double‐rows. Under superior point loading, no significant differences were observed for the rotational or translational outcomes. The repair with one double‐row demonstrated a significantly lower failure load than the repair with two double‐rows (388 N [243–463 N] vs. 602 N [539–641 N], *p* = 0.016, *q* = 0.031). No significant differences in stiffness were found between groups.

**Conclusions:**

Fixation with two double‐rows resulted in a significantly less fragment micromotion and a significantly greater failure strength compared with the BBB using one double‐row.

**Level of Evidence:**

N/A.

**Abbreviations**
BBB: bony Bankart bridge
PMMA: polymethyl methacrylate

## INTRODUCTION

Anterior shoulder dislocations may lead to anterior glenoid rim fractures due to the forces applied to the glenoid by the humeral head [3]. These bony Bankart fragments are found in 21% of patients with anterior shoulder dislocations [13]. If left untreated, fragment resorption may occur over time [21, 24], which may not only increase the risk of instability recurrence [17], but also complicate subsequent treatment if instability recurs [1, 5, 8, 32, 36]. Consequently, arthroscopic fragment refixation has been recommended, with good to excellent outcomes reported [4, 6, 10, 16, 18, 23, 26, 28, 29, 30, 33].

An arthroscopic single‐row refixation technique was published by Porcellini et al. more than 20 years ago with excellent outcomes reported [28, 29, 30]. In 2009, Millett et al. proposed a double‐row suture‐fixation of a bony Bankart injury called bony Bankart bridge (BBB) that utilizes a two‐point fixation that would allow for a stronger fixation and avoid tilting of the fragment to a greater degree than a single‐point fixation [22]. Previous biomechanical studies have reported more favourable biomechanical properties for the BBB when compared to the single‐row repair [9, 12, 35].

The BBB has been used and assessed both with a single or two rows (two or four anchors for fragment refixation, respectively), but it is yet unclear to what extent micromotion and failure load of the fixation would be altered through adding a second row. While previous studies have compared single‐ and double‐row repairs, the specific effect of adding a second double‐row on three‐dimensional fragment micromotion has not been previously quantified. The purpose of the present study was to assess micromotion and failure load when the BBB is performed with one versus two rows. It was hypothesized that micromotion would decrease and failure load would increase significantly when a second row is added.

## MATERIALS AND METHODS

### Specimen preparation

Seven pairs of fresh‐frozen cadaveric shoulders (median age: 54; mean age: 48 years [range, 22–65 years], sex: 57.1% male) were tested. Commencement of this study was exempt from institutional review board approval. Specimens with severe glenohumeral arthritis or injuries to the glenoid or labrum, as well as those with previous shoulder surgery, were excluded.

Specimens were thawed for 24 h prior to dissection and testing. For the dissection, skin, subcutaneous tissue and muscle were removed. The glenohumeral joint capsule was sharply dissected and the humerus was disarticulated. The remaining glenohumeral joint capsule, the coracoid and the long head of the biceps tendon just distal to its insertion were removed. Finally, the scapula was potted in polymethyl methacrylate (PMMA) within 5 cm of the glenoid in a mediolateral direction and at a 45° angle between the anterior glenoid and the anterior border of the potting.

### Study design

This study employed a paired study design. In seven randomly assigned shoulder specimens, one side of each of the seven pairs was randomly assigned to a treatment.

### Biomechanical testing and data collection

The biomechanical testing protocol was designed to compare micromotion and fixation failure load between the repair with one versus two double‐rows.

**Figure 1:**
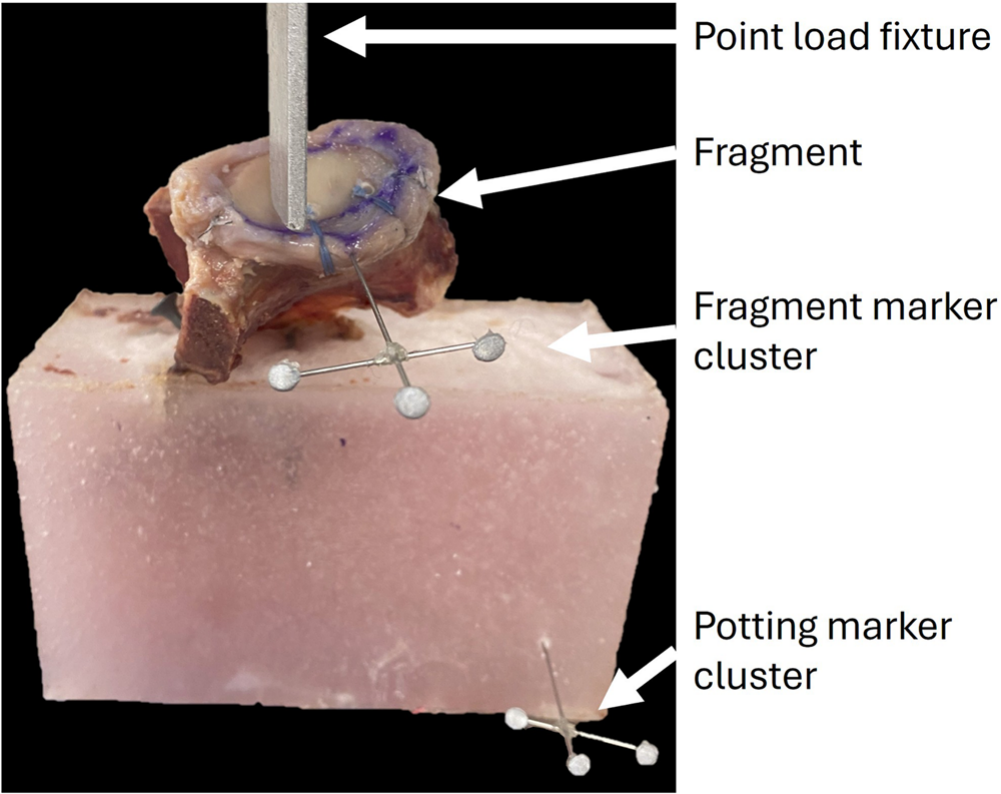
Mechanical testing setup.

Micromotion of the fragment was assessed with point loading applied to the superior and inferior thirds of the fragment in a random order. These loading locations were selected to apply controlled localized loading to two distinct regions of the fragment while minimizing confounding loading patterns. Loading the superior and inferior regions separately enabled assessment of regional differences in fixation stability while providing a comprehensive evaluation of three‐dimensional fragment micromotion relative to the glenoid. The specimen was mounted to a dynamic tensile testing machine (ElectroPuls® E10000; Instron®) with the potted scapula fixed to the base of the machine. A rectangular point load fixture of 3 mm thickness was secured to the end effector of the testing machine, and the specimen was aligned relative to the point load fixture such that a point compression load could be applied to either the superior or inferior third of the bony Bankart fragment (Figure 1).

**Figure 2:**
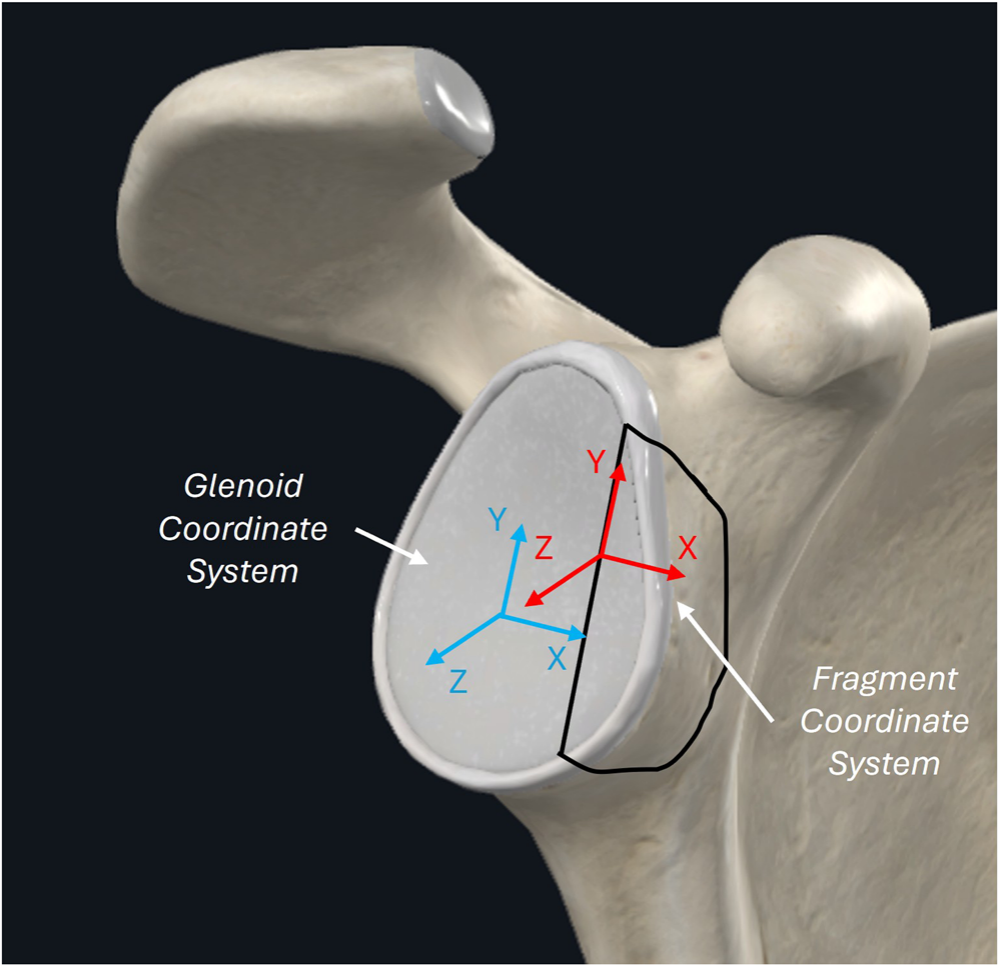
Schematic illustration of the glenoid and fragment coordinate systems.

To measure micromotion (relative translational and rotational microdisplacement between the bony Bankart fragment and the intact glenoid), coordinate systems were built for the fragment and the glenoid, clusters of three optical markers were fixed to the fragment and the glenoid and the optical system tracked the motion of the fragment coordinate system relative to the glenoid coordinate system. One marker cluster of three markers was secured to the fragment and another marker cluster of three markers was secured to the potting. Using a three‐dimensional digitizer (ROMER Absolute Arm; Hexagon AB), a glenoid‐centred coordinate system was created as described by Ohl et al. [25]. Twenty points were digitized around the glenoid rim. An ellipse was fit to the glenoid rim with the origin of the coordinate system located at the centre of the ellipse. The *z*‐axis was perpendicular to the least squares plane fitted to the glenoid cavity. The y‐axis was defined as the vector between the inferior and superior borders of the glenoid, projected on the glenoid plane and oriented superiorly. The *x*‐axis was orthogonal to both the *z*‐axis and *y*‐axis. The fragment location was digitized relative to the glenoid by marking the superior and inferior margins of the glenoid‐fragment interface. The fragment coordinate system shared the same *z*‐axis as the glenoid coordinate system. The *y*‐axis of the fragment coordinate system is defined as the vector between the inferior and superior margins of the glenoid‐fragment interface. The *x*‐axis of the fragment coordinate system is orthogonal to both the *z*‐axis and *y*‐axis, see Figure 2. Finally, the locations of the markers of each marker cluster were digitized.

To ensure contact between the point load fixture and the specimen, the tensile dynamic testing machine applied a nominal 2 N load to either the inferior or superior third of the glenoid rim of the fragment; the initial position of the end effector was recorded and the positions of the marker clusters were recorded. A cyclic loading protocol was applied to the glenoid rim from 5 to 20 N of compression at 1 Hz for 50 cycles. This loading range was selected to apply a controlled, low‐magnitude cyclic compressive force to the fragment while avoiding premature construct failure. The purpose of this loading protocol was not to replicate physiologic glenohumeral joint forces, but rather to provide a standardized loading condition that would allow quantification of fragment micromotion and enable comparison of fixation stability between repair constructs. Movement of the optical markers was captured via an optical motion tracking system (Qualisys Track Manager; Qualisys). Using a custom script (MATLAB; MathWorks) and the digitized points at each time point, the locations of the fragment marker clusters were expressed in the fragment coordinate frame and the locations of the glenoid marker clusters were expressed in the glenoid coordinate frame. Using singular value decomposition, a transformation matrix was calculated between the fragment frame at the current time point and the fragment frame from the initial time point. Relative translational displacement was calculated in the glenoid frame as the change in position of the origin of the fragment coordinate frame from the initial position to the current position. Relative rotational displacements about each axis of the glenoid frame were then extracted from the rotational portion of the transformation matrix. Upon completion of the cyclic loading protocol, failure testing was performed. The point load fixture was removed from the end effector and replaced with a metal tab with the same dimensions as the fragment. The tab was positioned such that the load was only applied to the fragment. Point loading was used as a controlled, non‐physiologic method to assess relative fragment micromotion under localized loading conditions. Ramp displacement was performed at a rate of 0.5 mm/s until the fragment reached failure. The failure load, defined as the point of maximum load, was recorded, and stiffness was calculated as the slope of the linear region of the load‐elongation curve [2]. After testing, the specimen was removed from the testing machine and examined, and the mode of failure was recorded.

### Creation of the bony Bankart lesion/Repair techniques

The glenoid surface was marked at its point of maximum width. A digital caliper was used to measure the bony length, and the anterior glenoid surface was then marked at exactly 25% of that measurement. A small bone saw was carefully used to make an isolated cut at the marked area, maintaining a 90° angle to the floor, without damaging the labrum.

**Figure 3:**
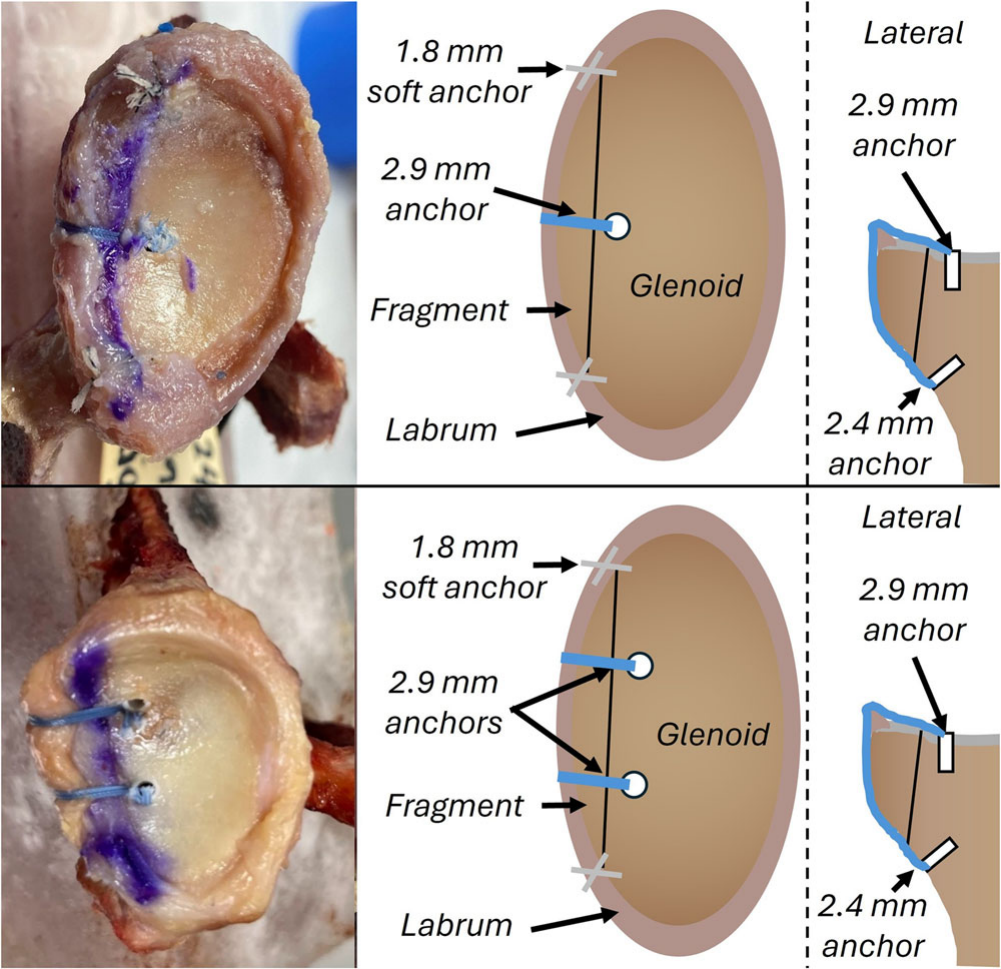
Images of the one double‐row (top) and the two double‐row repairs (bottom).

Images of the repairs can be found in Figure 3. The BBB was performed either using one or two double‐rows and, as previously described [10, 16, 22, 23], with the exception that the BBB was completed first and the labral repair was added thereafter. For the repair with one double‐row, a 2.4 mm anchor (BioComposite SutureTak® anchor; Arthrex Inc.) was placed medially on the glenoid neck and in the midportion of the fragment, and its sutures were passed around the fragment and the labrum. When two double‐rows were performed, two anchors were placed at the inferior and superior third of the fragment. The distance between the two medial anchors was individualized and determined by the longitudinal length of the glenoid surface. The anchors were brought in at the 33% and 67% points of the length. Laterally, each suture pair is fed into an anchor (2.9 mm PushLock®; Arthrex Inc.), which is then pushed into a drill hole on the glenoid face (one PushLock® anchor for the repair with one double‐row and two PushLock® anchors for the repair with two double‐rows). Afterwards, a labral repair was performed using a soft anchor (1.8 mm Knotless FiberTak® soft anchor; Arthrex Inc.) each inferiorly and superiorly to the bony Bankart lesion.

### Statistical analysis

A power analysis was performed using the mean and standard deviation of fragment‐glenoid motion toggle as reported by Greenstein et al. [12], as toggle motion is defined as two‐dimensional micromotion, which is most similar to the three‐dimensional micromotion outcome that was studied in this experiment. It was determined that seven pairs of specimens would be required (14 specimens in total) (*β* = 0.8, *α* = 0.05). Statistical analyses were performed using commercial software (MATLAB; MathWorks). Ten outcome variables were assessed: maximum cyclic rotation of the fragment (about the glenoid anterior–posterior axis, superior–inferior axis and medial–lateral axis), maximum cyclic translation of the fragment (along the glenoid anterior–posterior axis, superior–inferior axis and medial–lateral axis), failure load and repair stiffness.

Given the paired cadaveric study design and small sample size, data are presented as median and interquartile range (IQR) and paired comparisons between repair groups were performed using Wilcoxon signed‐rank tests. To account for multiple comparisons, Benjamini–Hochberg false discovery rate correction was applied separately within each outcome family: inferior loading micromotion outcomes, superior loading micromotion outcomes and failure‐testing outcomes. Raw *p* values and Benjamini–Hochberg‐adjusted *q* values are reported. Statistical significance was defined as *p* < 0.05.

## RESULTS

### Fragment micromotion

**Figure 4:**
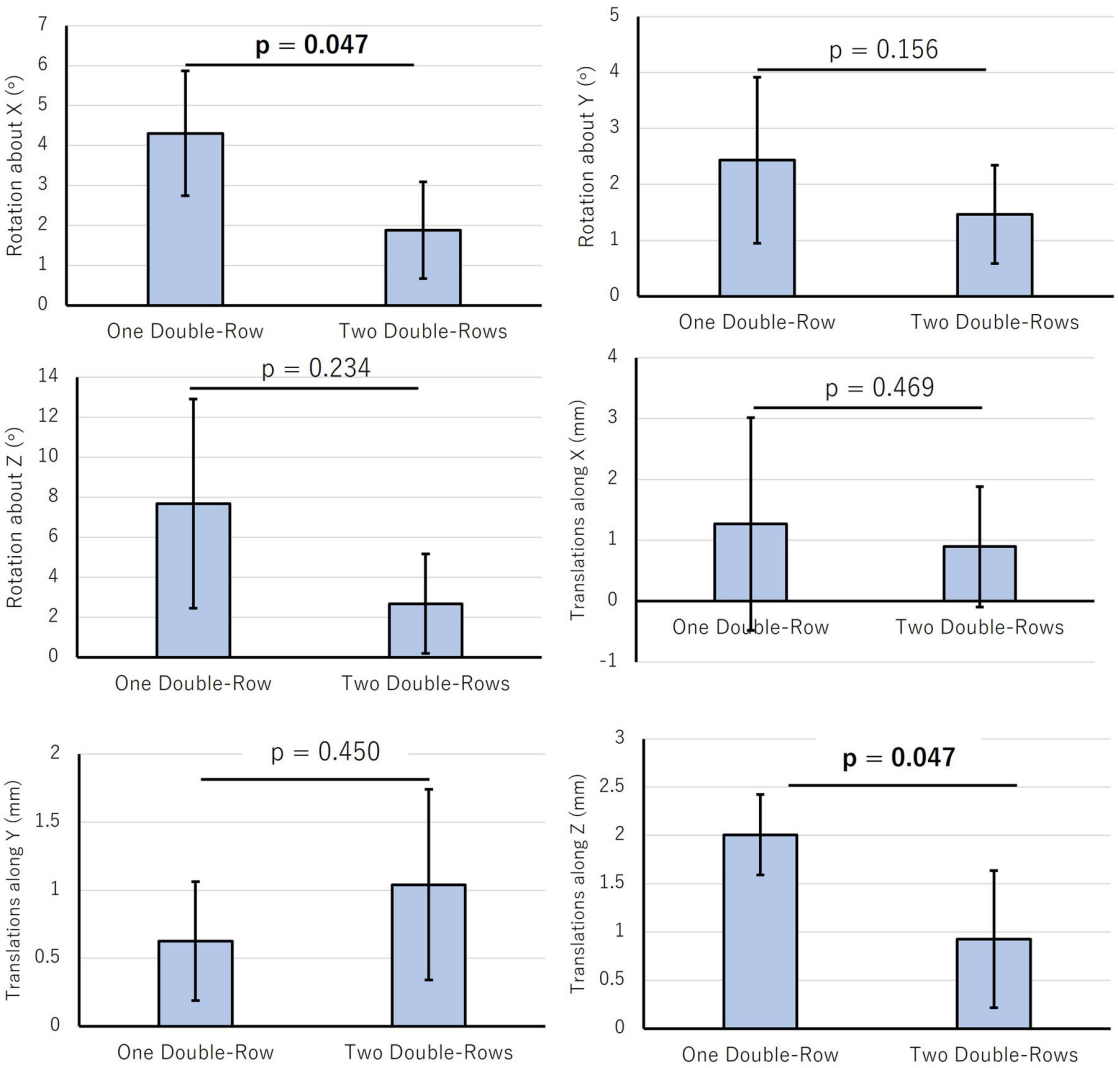
Micromotion data when an inferior load was applied to the fragment, including rotation about the *x*‐axis (top left), rotation about the *y*‐axis (top right), rotation about the *z*‐axis (middle left), translation along the *x*‐axis (middle right), translation along the *y*‐axis (bottom left) and translations along the *z*‐axis (bottom right).

**Figure 5:**
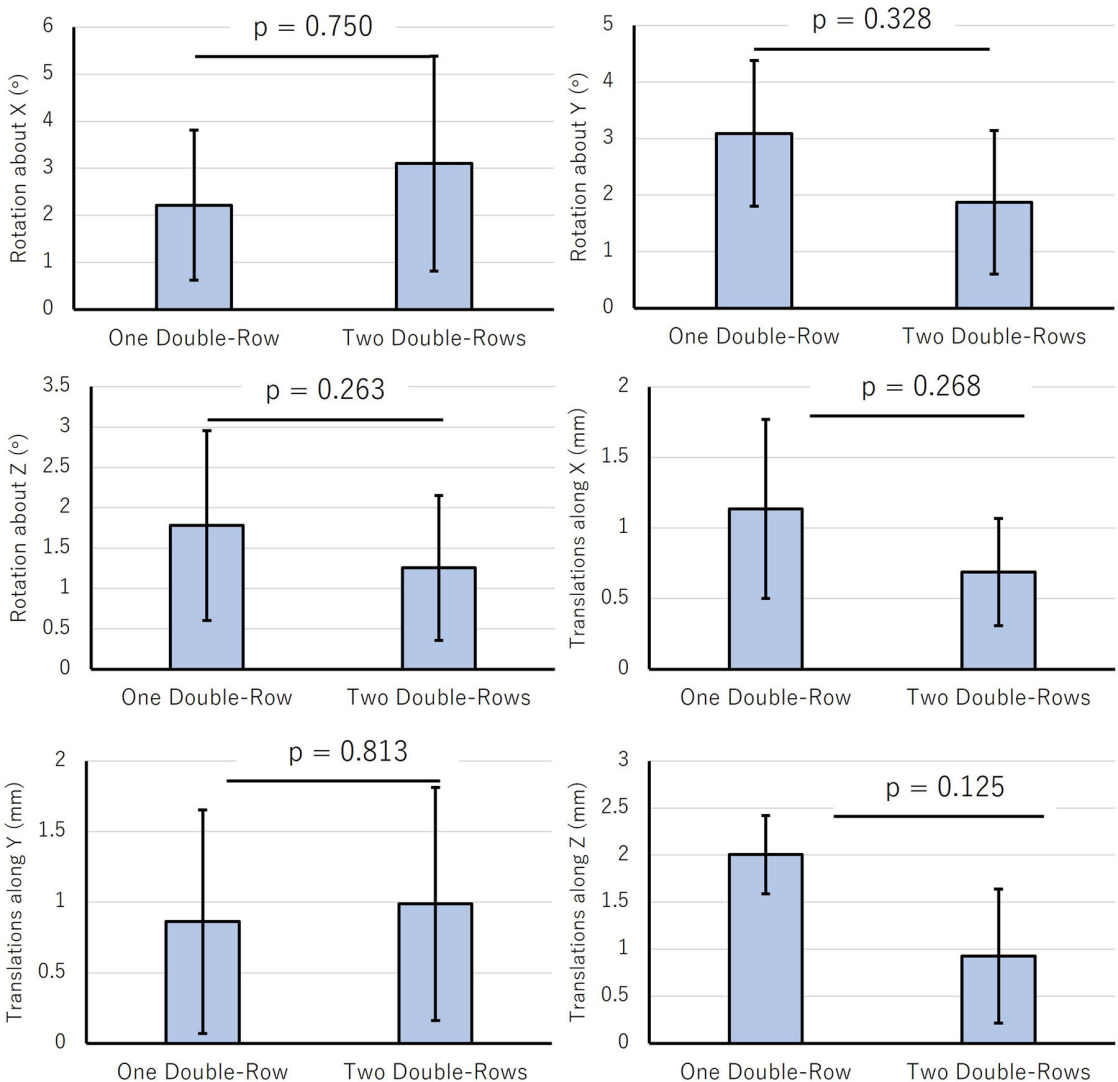
Micromotion data when a superior load was applied to the fragment, including rotation about the *x*‐axis (top left), rotation about the *y*‐axis (top right), rotation about the *z*‐axis (middle left), translation along the *x*‐axis (middle right), translation along the *y*‐axis (bottom left) and translations along the *z*‐axis (bottom right).

Fragment micromotion during the inferior and superior point loads is displayed graphically in Figures 4 and 5, respectively.

When an inferior point load was applied to the fragment, the repair with one double‐row demonstrated significantly greater rotation about the *x*‐axis than the repair with two double‐rows (5.20° [IQR, 3.01°–5.29°] vs. 1.64° [IQR, 0.94°–2.40°], *p* = 0.016, *q* = 0.047). The repair with one double‐row also demonstrated significantly greater translation along the z‐axis than the repair with two double‐rows (2.78 mm [IQR, 1.81–4.20 mm] vs. 1.06 mm [IQR, 0.51–1.51 mm], *p* = 0.016, *q* = 0.047). There were no significant differences regarding rotation about the *z*‐axis, translation along the *x*‐axis or both translation or rotation about the *y*‐axis between the two repair groups.

When a superior point load was applied to the fragment, translation along the *z*‐axis was greater in the repair with one double‐row than in the repair with two double‐rows (1.91 mm [IQR, 1.64–2.36 mm] vs. 0.73 mm [IQR, 0.40–1.41 mm], *p* = 0.031, *q* = 0.125), but this did not remain significant after correction. No other rotational or translational outcome demonstrated a significant difference between groups.

### Failure testing

**Figure 6:**
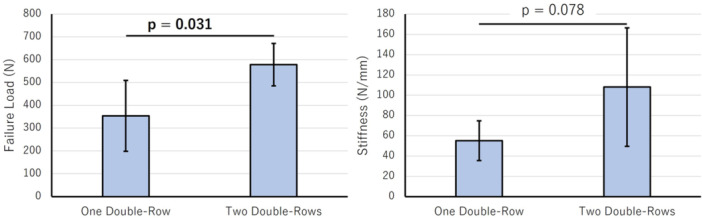
Failure load (left) and stiffness (right) comparison between the one double‐row repair and the two double‐row repair.

The repair with one double‐row demonstrated a significantly lower failure load than the repair with two double‐rows (388 N [IQR, 243–463 N] vs. 602 N [IQR, 539–641 N], *p* = 0.016, *q* = 0.031). There were no significant differences in linear stiffness between groups (53 N/mm [IQR, 43–69 N/mm] vs. 115 N/mm [IQR, 89–138 N/mm], *p* = 0.078, *q* = 0.078), see Figure 6. All specimens failed via suture pull‐through.

## DISCUSSION

The most important finding of the present study was that fragment micromotion was significantly reduced and failure load significantly increased when a second double‐row was added to the BBB. Significant reductions were observed for rotation about the *x*‐axis and translation along the *z*‐axis during inferior loading as well as translation along the *z*‐axis during superior loading. While not all translational and rotational degrees of freedom demonstrated statistically significant differences, the direction of effect was consistently favourable for the repair with two double‐rows across nearly all measured outcomes. Given that these variables represent related components of three‐dimensional fragment motion, isolated axis‐specific findings should not be interpreted independently but rather as part of an overall pattern indicating improved resistance to fragment displacement.

Previous biomechanical studies evaluating the BBB compared this technique to a single‐row repair [9, 12, 35]. Spiegl et al. [35] reported significantly superior stability and improved fracture reduction for the BBB compared to a single‐row repair. Giles et al. [9] found no significant difference between the BBB and a single‐row repair regarding failure load, glenoid load transfer and glenohumeral joint contact, but significantly less fragment displacement with the BBB during both centralized as well as eccentric loading. Greenstein et al. [12] reported a significantly smaller step‐off between the fragment and the glenoid as well as both less motion toggle and ultimate interface displacement for the BBB compared to a single‐row. In essence, it has been shown that the BBB is associated with superior biomechanical properties compared to a single‐row repair, but it was yet unclear to what degree the repair would benefit from a second double‐row. In the present study, failure load was significantly increased and translation along the *z*‐axis as well as rotation around the *x*‐axis, which would resemble fragment medialization or rotation at the glenoid‐fragment interface, were significantly reduced by adding a second double‐row. These biomechanical improvements may enhance fracture healing and stability while accelerating; however, they may be offset by longer operative times, more complex suture management and a potentially elevated risk of iatrogenic glenoid fractures [37]. Further, there exists a concern for chondral damage on the humeral head over time due to the intraarticular passage of sutures, which is already amplified in the BBB when compared with a single‐row repair [11]. Chondral damage may be further exacerbated when a second double‐row is added to the BBB. Patients who underwent the BBB with one or two double‐rows, however, did not need to undergo surgery for osteoarthritis and had little pain at a minimum 10‐year follow‐up [16], indicating that the findings of the above‐mentioned biomechanical study may be of limited clinical relevance. A good to excellent functional outcome, an overall recurrence rate of 11.9% and a return to sports rate of 91.0% with no clear trend favouring a single refixation technique were reported in a recent systematic review [6].

The magnitude of fragment micromotion is a critical determinant of bone healing. Mechanobiological and fracture‐healing studies have demonstrated that controlled cyclic axial micromotion in the range of approximately 0.2–0.5 mm promotes callus formation and osseous healing, whereas larger magnitudes of motion increase interfragmentary strain and favour fibrous tissue formation [14, 31]. In the context of a thin glenoid rim fragment with limited contact area, relative micromotion approaching 2 mm likely results in strain levels that are unfavourable for direct bone healing, particularly when motion includes rotational or shear components. While the precise micromotion threshold for successful healing of bony Bankart lesions remains undefined, these data suggest that millimetre‐scale differences in fragment stability may be biologically meaningful for early healing potential at time zero.

Several limitations should be considered when interpreting the findings of this study. First, this study assessed the biomechanical properties of two variations of the BBB at *t* = *0*. Consequently, it cannot speak to the impact the addition of a second double‐row would have on biological healing [26] as well as clinical outcome. This may, however, be evaluated in future clinical studies. Second, testing was performed using a point load fixture that applied force to the fragment, which may not replicate physiological loading. A concavity‐compression model evaluating stability through range of motion may have better emulated physiological conditions [12]. Further, while a labral repair was performed, overall capsulolabral tension, which may have affected the outcomes [15, 20], was not accounted for. Third, several relevant patient‐ and injury‐specific factors which may confound the results, such as overall capsular volume [27], hyperlaxity [5, 39] and the presence or extent of a Hill–Sachs lesion [5, 7, 19, 34, 38, 39] could not be accounted for.

## CONCLUSION

Fixation with two double‐rows resulted in a lesser fragment micromotion and a greater failure strength compared with the BBB using one double‐row.

## AUTHOR CONTRIBUTIONS

Maximilian Hinz contributed to ideation, project management, data collection, manuscript preparation and manuscript review. Ayham Jaber contributed to ideation, project management, data collection, manuscript preparation and manuscript review. Justin F. M. Hollenbeck contributed to ideation, project management, data collection, manuscript preparation, data processing, data analysis and manuscript review. Rio R. Castro contributed to data collection, manuscript preparation and manuscript review. Wyatt H. Buchalter contributed to data collection, manuscript preparation and manuscript review. Matthew T. Provencher contributed to ideation, project management and manuscript review. Peter J. Millett contributed to ideation, project management and manuscript review.

## FUNDING INFORMATION

Arthrex: IIRR‐02006Arthrex Inc.

## CONFLICT OF INTEREST STATEMENT

Maximilian Hinz, Ayham Jaber, Justin F. M. Hollenbeck, Wyatt H. Buchalter and Rio R. Castro report that their positions are supported by the Steadman Philippon Research Institute, which is a 501(c)(3) non‐profit institution supported by private donations and corporate funding. The Steadman Philippon Research Institute (SPRI) exercises special care to identify any financial interests or relationships related to research conducted here. During the past calendar year, SPRI has received grant funding or in‐kind donations from Arthrex, Canon, DJO, Icarus Medical, Medtronic, Ossur, Smith&Nephew, SubioMed and Stryker & Wright Medical. Matthew T. Provencher is an editorial board or governing board member for SLACK, Inc., a board or committee member for Arthroscopy Association of North America (AANA), American Academy of Orthopaedic Surgeons (AAOS), American Orthopaedic Society for Sports Medicine (AOSSM), American Shoulder and Elbow Surgeons (ASES), San Diego Shoulder Institute (SDSI) and Society of Military Orthopaedic Surgeons (SOMOS), serves on the medical board of trustees for the Musculoskeletal Transplant Foundation (through 2018); and declares royalties from Arthrex, Inc. Arthrosurface, Responsive Arthroscopy (2020) and Anika Therapeutics, Inc., consulting fees from Arthrex, Inc., Joint Restoration Foundation (JRF), Zimmer Biomet Holdings and Arthrosurface, grants from the Department of Defence (DoD), the National Institute of Health (NIH), the DJO (2020) and honoria from Flexion Therapeutics. Peter J. Millett has received IP royalties, consulting fees, and research support from Arthrex; research support from Ossur, Siemens and Smith & Nephew; and publishing royalties and financial or material support from Springer; and holds stock or stock options in VuMedi.

## ETHICS STATEMENT

The authors have nothing to report.

## Data Availability

Research data are not shared.
